# CountShoots: Automatic Detection and Counting of Slash Pine New Shoots Using UAV Imagery

**DOI:** 10.34133/plantphenomics.0065

**Published:** 2023-07-10

**Authors:** Xia Hao, Yue Cao, Zhaoxu Zhang, Federico Tomasetto, Weiqi Yan, Cong Xu, Qifu Luan, Yanjie Li

**Affiliations:** ^1^ College of Information Science and Engineering, Shandong Agricultural University, No. 61, Daizong Road, Taian 271018, Shandong Province, China.; ^2^ AgResearch Ltd., Christchurch 8140, New Zealand.; ^3^ Department of Computer Science, Auckland University of Technology, Auckland 1010, New Zealand.; ^4^ School of Forestry, University of Canterbury, Private Bag 4800, 8041 Christchurch, New Zealand.; ^5^ Research Institute of Subtropical Forestry, Chinese Academy of Forestry, No. 73, Daqiao Road, Fuyang, Hangzhou 311400, Zhejiang Province, China.

## Abstract

The density of new shoots on pine trees is an important indicator of their growth and photosynthetic capacity. However, traditional methods to monitor new shoot density rely on manual and destructive measurements, which are labor-intensive and have led to fewer studies on new shoot density. Therefore, in this study, we present user-friendly software called CountShoots, which extracts new shoot density in an easy and convenient way using unmanned aerial vehicles based on the YOLOX and Slash Pine Shoot Counting Network (SPSC-net) models. This software mainly consists of 2 steps. Firstly, we deployed a modified YOLOX model to identify the tree species and location from complex RGB background images, which yielded a high recognition accuracy of 99.15% and 95.47%. These results showed that our model produced higher detection accuracy compared to YOLOv5, Efficientnet, and Faster-RCNN models. Secondly, we constructed an SPSC-net. This methodology is based on the CCTrans network, which outperformed DM-Count, CSR-net, and MCNN models, with the lowest mean squared error and mean absolute error results among other models (i.e., 2.18 and 1.47, respectively). To our best knowledge, our work is the first research contribution to identify tree crowns and count new shoots automatically in slash pine. Our research outcome provides a highly efficient and rapid user-interactive pine tree new shoot detection and counting system for tree breeding and genetic use purposes.

## Introduction

In southern China, the slash pine (*Pinus elliottii*) is an exotic pine species, which has become one of the most domesticated tree species mainly for timber and resin production [1,2]. One key reason for the domestication of slash pine is its genetic improvement [3], which ensures sustainable wood timber and resin production and enables the selection of productive breeds through constant monitoring of growth traits. One of the most important traits, new shoot density, i.e., the number of new shoots per tree, should be one of the main focuses in the tree breeding strategy. The new shoots of pine trees play an important role in tree growth, nutrition accumulation, and retranslocation ([4]). Shoot density is closely associated with nutrient absorption and retranslocation, tree growth, crown size, and crown photosynthetic capacity, which could be an important indicator for the selection of timber and resin yield of productive tree [5–7].

A standard way to measure the new shoot density of pine trees is to count in each tree, which relies on human observation for the tree breeding process that is inefficient and time-consuming [8]. In addition, due to the tree height and crown density, it is difficult to count the new shoot in an efficient way. Therefore, most studies so far have focused on the length of new shoots [9] and less on new shoot density. To the best of our knowledge, the tree new shoot density and the absolute number have been less studied [10,11], and these studies primarily relied on manual counting methods, lacking an efficient and automated approach for quantifying shoot density and absolute numbers. It is therefore needed to develop automated new shoot detection techniques based on deep learning (DL) methodology to meet the modern requirement of high-throughput and efficient measurement of tree traits.

With the development of inexpensive and highly efficient unmanned aerial vehicles (UAV) camera platforms, in-field RGB image-based target detection has successfully emerged as a powerful and reliable solution to substitute laborious traditional manual measurement in agriculture and forestry studies [12–14]. UAV-based high definition RGB imagery holds the advantage in obtaining high-resolution target images in a high-throughput way and produces a relatively high accuracy coupled with the DL methodology [15]. The main process involves using a computer-based system to simulate human visual functions in order to detect relevant feature information from RGB images, ultimately achieving high-accuracy detection of plant targets. [16]. It has been successfully applied to the detection of the wheat head [17], ornamental plant [18], wheat yellow rust [19] and Gramineae weed detection [20].

The rapid development of computer software and hardware has dramatically improved the performance of computer vision technology for the application of various plant traits. Machine learning (ML) and DL methods are the 2 mainstream methods for plant trait counting. ML, such as support vector machines [21] and random forests [22], has been harnessed to build regression or classification models for plant target detection and counting by extracting features such as color and texture from images [23,24]. However, ML requires human-defined features, and as the data increases, the performance is saturated, which is not suitable for the growing demands of large data processing [25,26].

DL is a branch of ML that utilizes neural networks to process large amounts of data, reduce prediction errors, and automatically extract features [27,28]. It has been widely applied in plant trait detection to meet the requirement of accurate plant trait counting [29].

The mainstream counting method of DL uses convolutional networks to regress density maps, which often has difficulties to capture global features for global context modeling and usually requires the introduction of additional attention mechanisms, with the model structure that is gradually complicated [30]. Vision Transformer ([31]), with its powerful global context modeling capabilities, shows powerful processing capabilities in dense prediction tasks such as object detection and segmentation. Liang et al. [32] proposed TransCrowd, which reformulates the problem of weakly supervised population counting from the perspective of Vision Transformer-based sequence counting, is also the first model for weakly supervised population counting based on Transformer. Instead of generating predicted density maps, it trains directly from images to counts in a weakly supervised manner, based on the number of people in the images. Inspired by classification, Background Contextual Transformer [33] added a context token in the input sequence to enable better information exchange between the transformer in the model and image patches. Compared with the work of TransCrowd, the overall network processing method by Sun et al. [33] is simplified and better performance is achieved. Another counting model, CCTrans [34], also has been proposed, with its crowd counting model using Twins as the backbone network [35]. The feature pyramid fusion module is used in the CCTrans model to supplement detailed information with low-level features, resulting in crowd features that are rich in semantics, detailed information, and global features. Finally, a multiscale dilated convolution module is designed as the regression head to deal with the global features captured by the Transformer, which is very helpful in regressing a more precise and accurate crowd density map. This method optimizes the loss function of the mainstream with strong and weak supervised forms to improve the generalization performance and robustness of the model.

Therefore, in this paper, we propose to construct a Slash Pine Shoot Counting Network (SPSC-net) model based on CCTrans to count the new shoots of slash pine. We introduced the feature pyramid module to achieve accurate counting of slash pine new shoot. In summary, the aims of this paper are to (a) Construct a target extraction network to accurately detect and extract the individual slash pine trees from the complex background; (b) create a multiscale slash pine new shoot counting network SPSC-net, which is based on CCTrans and introduces nonequilibrium transmission and perspective guidance to improve the loss function in the fully supervised process. As a result, it efficiently and accurately completes the counting task in multiscale wetland pine images; (c) create a highly efficient user-interactive pine tree new shoot detection and counting system for the convenient of tree breeding and genetic use purpose.

## Materials and Methods

**Fig. 1.**
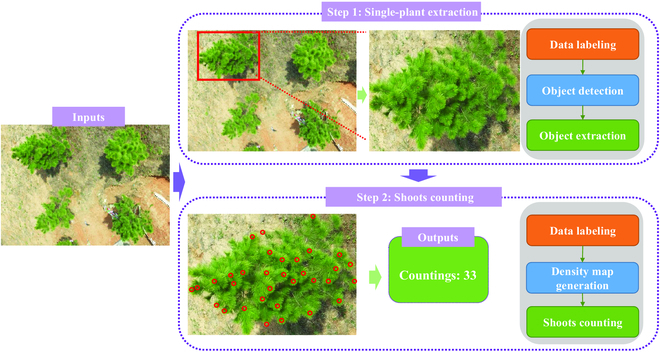
The overall research roadmap.

In this paper, we built a framework for automatically counting new shoots on UAV images. The UAV images containing one or more plants were used as input. The framework (Fig. 1) was developed in 2 stages. The first step is the extraction of a single plant, which builds a target extraction network based on a manually labeled data set to realize the detection and segmentation of a single slash pine, in order to obtain a single target and discard complex backgrounds. The second step is to count the new shoots. This method is needed to generate a density map based on the center coordinate points of the manually labeled tipping as an intermediate representation to supervise the learning of the counting model. The trained counting model can directly predict the number of shoots. More details are described in the following sections.

### Site location and images collection

**Fig. 2.**
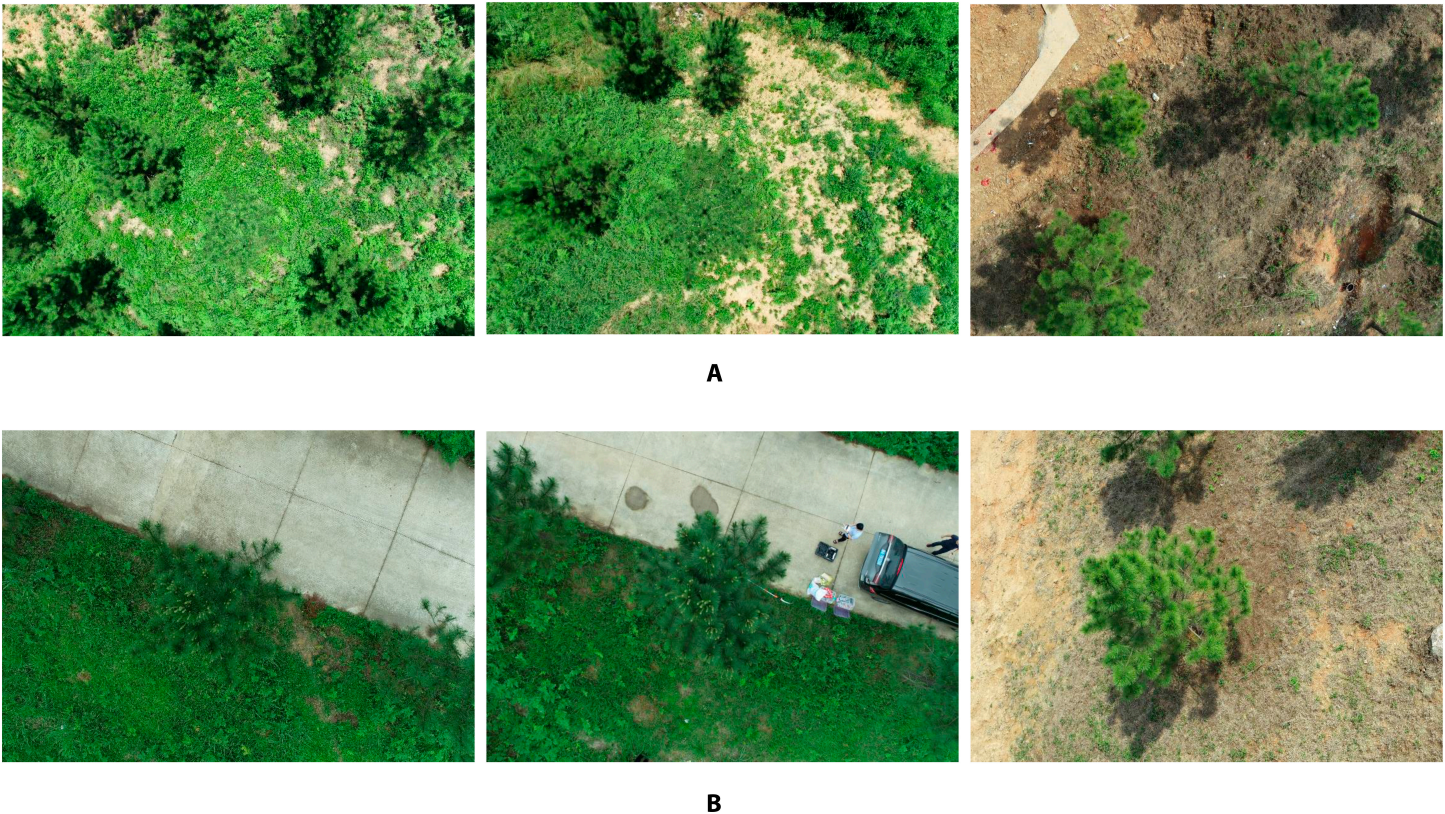
Examples of original UAV images. (A) With multiple slash pine trees. (B) With single slash pine trees.

The site for a breeding population of slash pine is located at a Matou national forest farm in Xuancheng, Anhui, China (30°45′N, 118°29′E), covering an area of approximately 49.4 acres. The information on this site was described by Song et al. [36]. In brief, this site is located in a subtropical climate, with precipitation and average yearly temperature of 1,520 mm and 15.7^°^C, respectively. The images of each slash pine tree were collected in March and June 2022, respectively, using DJI Phantom 4 RTK UAV with a 4,864 × 3,648 resolution RGB camera (DJI, Shenzhen, Guangdong, China). To obtain images of new shoots at different growth stages, 2 flights per month were conducted in both March and June, with an interval of about 10 d between each flight. To avoid the severe sunlight affecting the image quality, cloudy days were chosen for each flight. The flight altitude was set at 20 m at 90°, with side and front image overlap percentages of 80% and 85%, respectively. Each image has a resolution of 5,472 × 3,648 dpi. After removing images with severe blur and distortion, a total of 1,860 images were applied for data analysis. A subset of images of slash pine is shown in Fig. 2.

In both experiments (i.e., single-plant extraction and new shoots counting), the original dataset was divided into training, validation, and test sets in a ratio of 7:1.5:1.5. In the object detection task, the number of original images in the training, validation, and test sets were 1,302, 279, and 279, respectively. In the counting task, the numbers were 219, 47, and 47 for the training, validation, and test sets, respectively.

### Data labeling

**Fig. 3.**
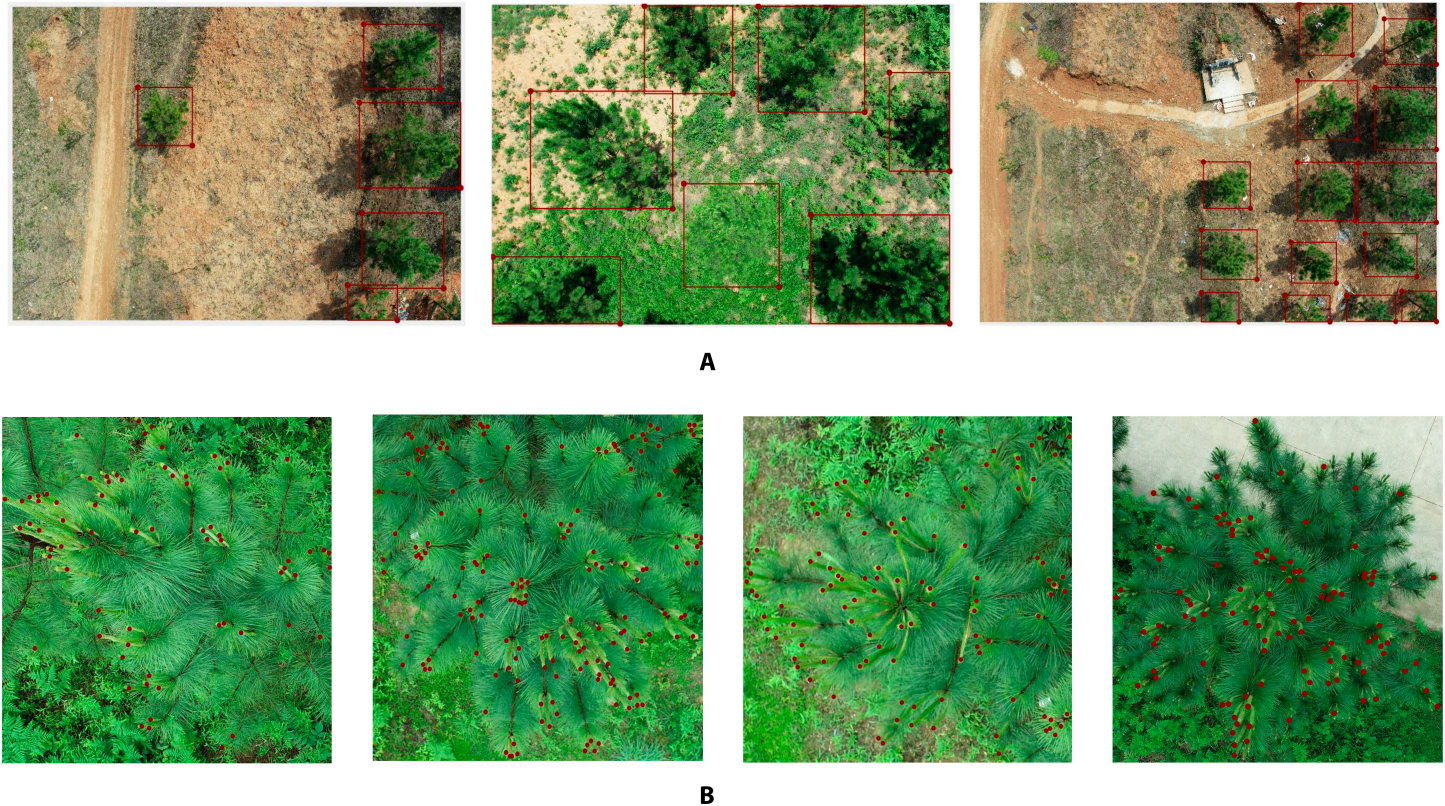
Example of data labeling. (A) Labeling of individual plant extracts. (B) Labeling of new shoots counting.

According to the 2 processes of shoot counting, 2 types of labeling were created, including every single tree and the new shoots in each tree (Fig. 3A and B). Each label in Fig. 3A records the position of the circumscribed rectangle, which is annotated with LabelImg [37]. The section involved labeling a total of 1,860 images and 8,488 bounding boxes. The labels in Fig. 3B show the point position of each new shoot along with the number of the whole plant, which is annotated by MATLAB. We tackled the issue of counting by labeling 313 images with 36,758 points.

### Density map generation

**Fig. 4.**
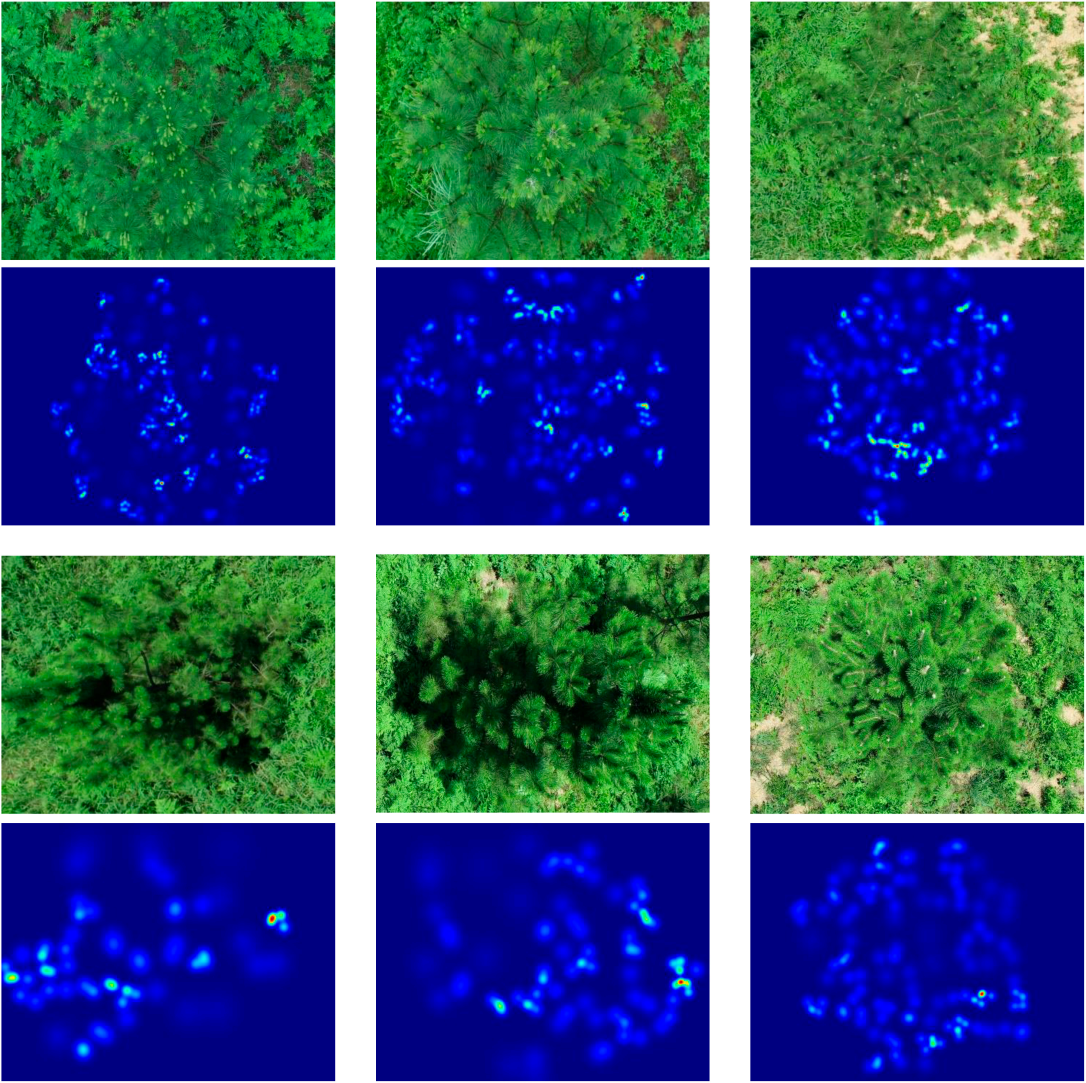
Samples of GT density map for counting labels.

The network can deal with complicated scenes and high-density tree crown overlapping by using the density map as the intermediate representation to supervise the learning of DL models. Therefore, a counting network based on a density map was designed. In order to map the point coordinates obtained by labeling the new shoots of slash pine into a true-value density map, a geometrically adaptive kernel method [38] was used. In Fig. 4, the dark blue part indicate the background, and the highlighted warm color area represents the densely distributed new shoots.

### Single-plant extraction network

We introduced a YOLOX algorithm [39] as the basic network structure to obtain the detection and extraction of single slash pine. This algorithm was adjusted according to the requirements of detection tasks, including backbone, neck, and head network, respectively. The backbone network of YOLOX followed the Darknet network of YOLOv3 [40]. Firstly, the model downsampled the input data through the focus structure to reduce the computational overhead required for model inference. The neck network of the plant extraction model was based on the image feature pyramid network [41]. This detects slash pine trees at different scales and accurately identifies the slash pine trees with overlapping. A neck network obtained the feature map of the extraction target from 3 different scales for regression analysis. This added an IoU branch into the regression branch to complete the extraction task of single slash pine. During the training process of the model, we set the epoch to 200 and the learning rate to 0.01.

### New shoots counting model

At present, the mainstream method for counting objects is convolutional neural network. However, due to the limited receptive field, it is difficult to capture global features for context modeling that is often necessary to introduce additional attention mechanisms [42], which also complicates the model structure [43]. Therefore, we constructed an SPSC-net. This methodology, which is based on the CCTrans network [34], generates a generalized loss function to learn the density map for shoot counting and location through the nonequilibrium optimal transfer. The main process is as follows:

For a fully supervised process, CCTrans is based on the design of the loss function proposed by Wang et al. [44], which is composed of a weighted sum of counting loss, optimal transport (OT) loss, and total variation (TV) loss. As the TV loss uses the original head annotation from ground truth (GT), which cannot be used to construct the segmentation target, the mean square error (*L*_2_) is used as a substitute for $L_{\mathrm{TV}}$. The original function treats the density map and the point lattice map as probability distributions and uses balanced OT to match the shapes of these 2 distributions. The point lattice map refers to a discrete grid or lattice representation, where each grid cell represents a specific location or region in the image space. Here, the point lattice map serves as a reference or target for the density map, enabling the comparison and alignment of their distributions using OT. For the predicted density map *D* and the GT *D*^′^, the total loss is calculated as follows:

$$L_{d}=L_{1}\left(P,G\right)+\lambda_{1}L_{\mathrm{OT}}+\lambda_{2}L_{2}\left(D,D^{\prime }\right)$$ (1)

where *P* and *G* denote the number of new shoots of *D* and *D*^′^, and *λ*_1_ and *λ*_2_ are the loss coefficients. The loss function *L*_1_, which corresponds to the smoothed *L*_*1*,_ is used to improve the robustness of the network. In addition, the smoothed annotation map, which refers to the density map after a smoothing process, is utilized to enhance the network's robustness. Furthermore, the mean square error (i.e., *L_2_*) is used to adjust the gap between the prediction and the smoothed annotation map. $L_{\mathrm{OT}}$ is the OT loss, which will be discussed in the following paragraph. One of the main shortcomings of the CCTrans network is that normalization of the density map predictions and point lattice maps for computing balanced OT removes the actual counts in both maps. This requires additional counting loss to ensure accurate prediction of counts, which is the sum of the density map. The balanced OT loss is used to align the predicted density map with the GT density map, while the counting loss is used to encourage accurate count predictions. The counting loss provides poor monitoring because its gradient adds the same outlier to all pixels, regardless of their relevance to the crowd count. This means that the loss may prioritize minimizing errors in unimportant regions, while neglecting errors in regions that are crucial for accurate counting. We proposed to consider nonequilibrium OT in our approach because it ensures that any discrepancies between the predicted and GT annotations are penalized. This is achieved by our pixels and point loss terms, which directly monitor incorrect predictions. Therefore, any imbalances in the process are accounted for and minimized during optimization. Formally, suppose the predicted density plot is:

$$A={\left\{\left(a_{i},x_{i}\right)\right\}}_{i=1}^{n}$$ (2)

Here, $a_{i}$ represents the predicted pixel density, and *x_i_ϵℝ*^2^ represents the number of pixels. We used *a* = [*a_i_*]*_i_* which represents the predict density map. The ground reality dot plot, represents the actual data points, can be shown as:

$$B={\left\{\left(b_{j},y_{j}\right)\right\}}_{j=1}^{m}$$ (3)

In a dot plot, each data point is represented by a dot on a 2-dimensional coordinate system, where *y_j_* is the position of the *j_th_* marking, *m* is the number of marking points, and *b_j_* is the number of target represented by the marking. This plot provides a visual representation of the spatial distribution and quantity of the targets in the dataset. In this study, we assumed: *b* = [*b_j_*]*_j_* = 1*_m_*, Our loss function was based on the optimal transmission cost of the imbalance of entropy regularization, as follows:

$$\begin{array}{cc} L_{C}^{T}\left(A,B\right)= & \min_{P\in {\mathbb{R}}_{+}^{n\times m}}\langle C,P\rangle -\varepsilon H\left(P\right)+\tau D_{1}\left(P1_{m}a\right)\\ & +\tau D_{2}\left(P^{T}1_{n}b\right) \end{array}$$ (4)

where $C\in {\mathbb{R}}_{+}^{n\times m}i$ is the transportation cost matrix; *P* is the transmission matrix, which will get the best shipping costs from A to B at each location by minimizing the treatment; 〈*C*, *P*〉 is the transmission loss, which pushes the predicted density toward the label during training. This loss term characterizes the difference between 2 distributions, similar to the OT loss in the original loss function. $H\left(P\right)=\sum_{\mathrm{ij}}P_{\mathrm{ij}}\text{log}P_{\mathrm{ij}}$ is the entropy regularization term, which encourages balanced or uniform distribution in the transportation scheme and is related to the unbalanced OT loss. When the value of ε is larger, the predicted density map becomes less compact. The TV loss term $\tau D_{1}\left(P1_{m}a\right)+\tau D_{2}\left(P^{T}1_{n}b\right)$ includes the product terms of matrices D1 and D2, as well as the matrix P normalized by rows and columns. *D*_1_ constrains the row sums of the transportation matrix P to match the GT point-annotated density map, while *D*_2_ provides auxiliary constraints to align the column sums of P with the reconstruction of GT point annotations. This complements the counting loss and further improves the accuracy of density map prediction, in which $\hat{a}$ = $P1_{m}$ is the intermediate density map representing the GT annotation construction; $\hat{b}$=$P^{T}1_{n}$ is the reconstruction of the GT point annotation. Compared to fixed transport with fixed parameters, adaptive transport adjusts automatically during the transportation process based on the network model, enabling more accurate prediction of density maps. During the experiment, *ε* was set to 0.05 and *τ* was set to 0.5.

In view of the problem that TV loss is prone to overfitting, we used squared L2-norm (‖.‖_2_) for the pixel-wise term and L1-norm (‖.‖_1_) for the point-wise term:

$$D_{1}\left(P1_{m}a\right)={\left\|P1_{m}-a\right\|}_{2}^{2}$$ (5)

$$D_{2}\left(P^{T}1_{n}b\right)={\left\|P^{T}1_{n}-b\right\|}_{1}$$ (6)

In addition, the original loss used the standard square Euclidean distance as the transportation cost, equally considering all distances. Considering the perspective effect in the slash pine shoot image, the new shoots close to the drone will appear sparser, and the new shoots further away from the UAV will appear dense in the image. In order to better represent the density of the new shoot at a distance, the transportation cost of these areas should be higher. Therefore, this study proposed a perspective-based transfer cost matrix to address the scale variation in the image. The cost function is defined as:

$$C_{\text{ij}}=\exp\left(\frac{1}{\eta \left(x_{i},y_{j}\right)}{\left\|x_{i}-y_{j}\right\|}_{2}\right)$$ (7)

where $\eta \left(x_{i},y_{j}\right)$ is the adaptive perspective coefficient. In order to adapt to the variations of various factors in different shooting scenes, the value of $\eta \left(x_{i},y_{j}\right)$ is determined as the average of the normalized heights of the 2 target pixels in their respective images, that is, $\frac{1}{2}\left(h_{x_{i}}+h_{y_{i}}\right)$. In addition, the Euclidean distance, represented by the cost function *L_ij_* = ‖*x_i_* − *y_i_*‖_2_, is used to measure the distance cost between 2 points. This formula is essentially exponentializing the original Euclidean distance divided by an adaptive perspective factor, which amplifies the transmission matrix above the image (often the wetland pine canopy) and further improves the accuracy of shoot counting.

### Model evaluation

#### Single tree detection model

Recall, precision, and recognition AP were employed as the evaluation indexes of the single tree extraction model. Recall is the proportion of all positive samples in the test set that are correctly identified as positive samples. Precision refers to the proportion of actual positive samples in the target detected as positive samples, with recall and precision as the horizontal and vertical coordinates to form a precision-recall (P-R) curve. The area under the entire P-R curve is AP: the higher AP is, the better its detection performance. The recall, precision, and AP equations are as:

$$\text{Recall}=\frac{\mathrm{TP}}{\mathrm{TP}+\mathrm{FN}}$$ (8)

$$\text{Precision}=\frac{\mathrm{TP}}{\mathrm{TP}+\mathrm{FP}}$$ (9)

$$\mathrm{AP}=\int_{0}^{1}p\left(r\right)\mathrm{dr}$$ (10)

where TP represents the number of correctly detected detection, FP shows the number of detections detected by false detection, FN indicates the number of detection distinguished by leakage, and *p*(*r*) reveals the P-R curve, respectively.

#### New shoots detection and counting model

The mean squared error (MSE) and mean absolute error (MAE) were accommodated as evaluation indicators for the new shoots detection and counting model. MSE is generally used to detect the deviation between the predicted value and the true value of the model. MAE is the average of the absolute error, reflecting the actual error of the predicted value, calculated as follows:

$$\text{MSE}=\frac{1}{n}\sum_{i=1}^{n}\left|P_{i}-G_{i}\right|$$ (11)

$$\text{MAE}=\sqrt{\frac{1}{n}\sum_{i=1}^{n}{\left|P_{i}-G_{i}\right|}^{2}}$$ (12)

where *n* is the number of test images and *P_i_*, and *G_i_* indicate the predicted and actual number of new shoots in the *i*th image, respectively.

In order to prove the effectiveness of the SPSC-net used in this study, it was compared with other classical counting algorithms including DM-Count [45], CSR-net [46], and MCNN. The evaluation algorithm was set according to the environment and parameters of SPSC-net, the batch size was set to 16, and the initial learning rate was set to 10^(-5).

All of the analysis in this study was conducted using PyCharm 2021 and the PyTorch 1.11.0 DL framework. The computer ran 128G of memory, the CPU model is Inter (R) Core (TM) i7-10700k, equipped with NVIDIA GeForce RTX 3060, the operating system was Windows 10, and the CUDA version is 11.3.109.

## Results

### Slash pine tree detection

As shown in Table 1, the threshold, set to 0.5, is used as a criterion to determine the tree detection results. It serves as a threshold for the predicted probabilities, where predictions with probabilities above 0.5 are classified as trees. By setting the threshold to 0.5, the tree detection precision and recall of YOLOv5, Efficientnet, and YOLOX are approximately 90%. When the threshold increased to 0.75, precision, recall, and AP of YOLOX was the best in all models, with APs of 99.29% and 96.08%, respectively. In comparison, YOLOv5 performs slightly worse at higher thresholds but still outperforms Efficientnet and Faster-RCNN. Faster-RCNN performs the worst among all models, with recall, precision, and AP values all below 30% at the 0.75 threshold. For the purpose of objectively evaluating the detection performance of various models under the conditions of overlapping targets, blurriness, and complex background, a manual counting of the specific detection situation for 26 test images was conducted. Table 2 shows statistical information for 26 test images, including the total object count, the number of correctly detected objects, false detections, and missed detections. YOLOX and YOLOv5 showed a relatively low false detection rate. EfficientNet had the lowest number of missed targets, but the false detection rate was slightly higher, mainly due to some targets being detected repeatedly. Overall, YOLOX exhibited the best performance most correctly detected objects, while Faster-RCNN performed the worst in this regard.

**Table 1.** The model performance of different tree detection models.

Model	Threshold = 0.5			Threshold = 0.75		
	Recall	Precision	AP	Recall	Precision	AP
YOLOX	99.01%	90.21%	99.29%	96.21%	87.66%	96.08%
YOLOv5	96.84%	90.09%	98.16%	89.17%	82.96%	87.42%
Efficientnet	97.75%	87.43%	97.80%	92.81%	83.01%	91.20%
Faster-RCNN	71.20%	27.78%	57.53%	29.59%	11.55%	13.88%

**Table 2.** Partial object detection results of different models.

Image ID	Total boxes	YOLOX			YOLOv5			Efficientnet			Faster-RCNN		
		Correct detection	False detection	Missed detection	Correct detection	False detection	Missed detection	Correct detection	False detection	Missed detection	Correct detection	False detection	Missed detection
1	13	13	0	0	13	0	0	13	0	0	8	3	5
2	15	15	0	0	15	0	0	15	0	0	9	12	6
3	10	10	0	0	10	2	0	10	3	0	7	2	3
4	5	5	0	0	5	1	0	5	4	0	4	4	1
5	5	5	0	0	5	1	0	5	5	0	4	1	1
6	2	2	0	0	1	0	1	2	3	0	2	4	0
7	6	6	1	0	2	0	4	6	1	0	5	2	1
8	7	7	1	1	3	0	4	7	1	0	5	2	2
9	8	6	0	2	3	0	5	8	0	0	4	2	4
10	5	5	0	0	5	2	0	5	4	0	4	8	1
11	8	8	1	0	8	0	0	8	0	0	6	11	2
12	9	9	0	0	9	1	0	9	1	0	5	5	4
13	14	14	0	0	14	1	0	14	4	0	6	3	8
14	16	16	0	0	16	0	0	16	2	0	12	6	4
15	12	12	0	0	12	0	0	12	2	0	6	4	6
16	17	17	0	0	17	0	0	16	1	0	10	5	7
17	11	11	0	0	11	1	0	11	1	0	5	4	6
18	6	6	0	0	6	0	0	6	0	0	5	7	1
19	7	7	3	0	7	2	0	7	5	0	5	4	2
20	13	13	1	0	13	0	0	13	0	0	4	4	9
21	24	24	0	0	24	0	0	24	1	0	10	9	14
22	36	35	1	1	31	0	5	34	4	2	5	8	31
23	10	9	1	0	8	0	2	10	2	0	6	7	4
24	8	8	1	0	8	0	0	8	1	0	3	4	5
25	19	16	1	3	15	0	4	19	2	0	4	5	15
26	17	17	1	0	16	0	1	18	1	0	5	4	12
Sum	303	296	12	7	277	11	26	301	48	2	149	130	154

**Fig. 5.**
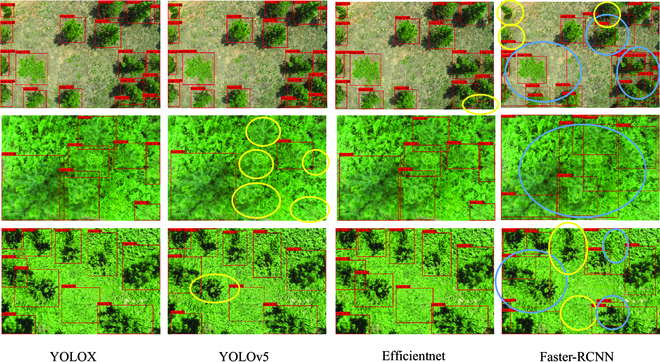
Detection effect of different models. The missed detections of the model are indicated by yellow circles, and false detections are indicated by blue circles.

Some results of the 4 models on the detection of slash pine trees canopy have been displayed in Fig. 5. It shows that YOLOv5, Efficientnet and Faster-RCNN have issues with missed detections (yellow marked area). Moreover, in dense tree crown areas, Faster-RCNN also suffers from marked problems with repeated detections (indicated by the blue marked areas). However, YOLOX shows the best detection results in complex backgrounds and overlapping targets.

### New shoots detection model

**Fig. 6.**
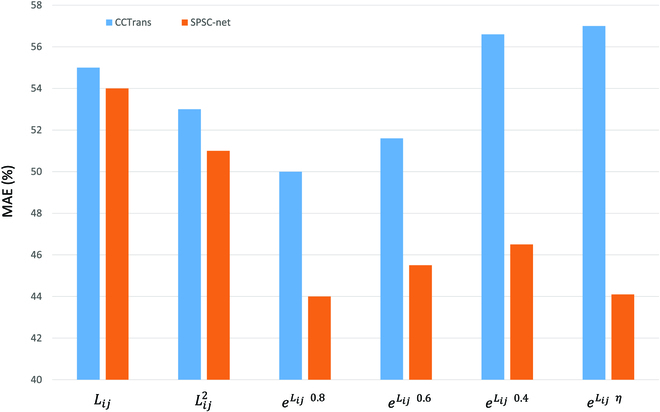
Comparison of different loss functions. The x-axis represents different cost functions, while the y-axis indicates the MAE results of the experiments.

The balanced OT of the original loss and the unbalanced OT framework in this study were compared. We evaluated the different transposition cost matrices (including Euclidean distance *L_ij_*, squared Euclidean distance *L^2^_ij_* and exponential Euclidean distance *e^L_ij_^*) and the effectiveness of the perspective factor value in the transmission cost matrix on MAE (Fig. 6). The base cost is Euclidean distance *L_ij_* = ‖*x_i_* − *y_i_*‖_2_. As clearly shown, the exponential function outperforms the traditional cost function based on Euclidean distance, while the perspective-guided model gets the best performance. This proves the efficacy of nonequilibrium OT for density regression problems. This directly draws the point-by-point loss and pixel loss to penalize additional/missing density, while the original loss requires additional counting loss, reducing counting efficiency.

In addition, the TV loss in the original loss is supervised pixel by pixel by using the normalized dot plot, which is prone to overfitting. The loss we used was more efficient at pushing density from the background to the annotation than the square Euclidean cost. The blue section represents the results of the original loss function of CCTrans, while the orange section represents the results of our improved loss function in SPSC-net.

**Fig. 7.**
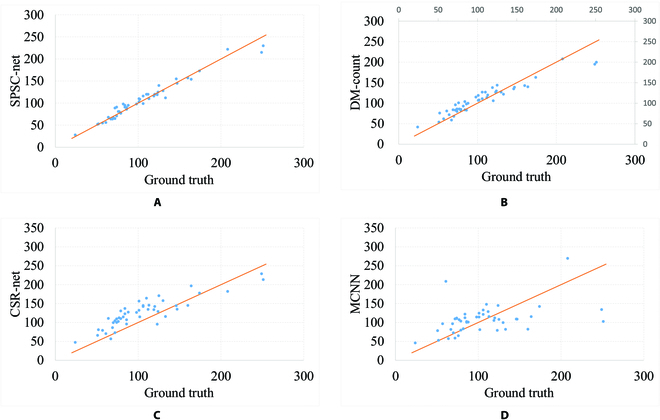
Counting results by different methods on test set. The x-axis showing the GT values and the y-axis indicating the predicted values. (A) Counting results of SPSC-net. (B) Counting results of DM-Count. (C) Counting results of CSR-net. (D) Counting results of MCNN.

The SPSC-net model had the lowest MSE and MAE among all the models, with values of 7.0 and 2.27, respectively (Table 3). Followed by the DM-Count and CSR-net, MCNN yielded the highest MSE and MAE results (34.50 and 30.76, respectively). Figure 7 illustrates the counting performance of SPSC-net, DM-Count, CSR-net, and MCNN on the test set through a scatter plot. The results demonstrate that SPSC-Net achieves high prediction accuracy, followed by DM-Count, while MCNN has the poorest prediction performance. DM-Count and CSR-Net often overestimate the count, leading to predicted values higher than the true values.

**Table 3.** Comparison of new shoots counting of different methods.

Model	MSE	MAE
SPSC-net	7.00	2.27
DM-Count	12.85	6.84
CSR-net	27.06	6.50
MCNN	31.12	30.76

**Fig. 8.**
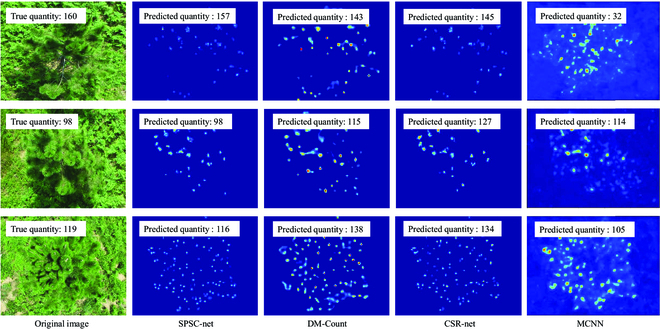
Density plot examples by different methods.

The performance of SPSC-net, DM-Count, CSR-net, and MCNN was visualized in Fig. 8. The predicted density map of different models based on the same image and the predicted number of new shoots, compared with the original image, showed that the experimental image using the SPSC-net model can clearly reflect the distribution of the drawbacks and its denseness (Fig. 8).

### Development of CountShoots application

In order to maximize the application value of the above model, we proposed to integrate and develop a slash pine extraction and new shoots counting system called CountShoots for forestry researchers. The system was implemented based on the Flask framework [47]. By loading the trained object detection model and counting model, the individual crown extraction and automatic counting of slash pine are sequentially realized. The functional modules of the system have been separated into user interaction module, model loading module, plant extraction model, and shoots counting module:

1. User interaction module, which is used to submit the image to be predicted and display the counting result.

2. The model loading module, which loads the trained slash pine plant extraction and counting models into memory for the next step of plant extraction and new shoots counting.

3. Plant extraction module, which extracts individual plants from the original UAV image using the trained plant extraction model (Single-plant extraction network).

4. The shoots counting module, which uses the shoots counting model to count the new shoots of individual slash pine.

**Fig. 9.**
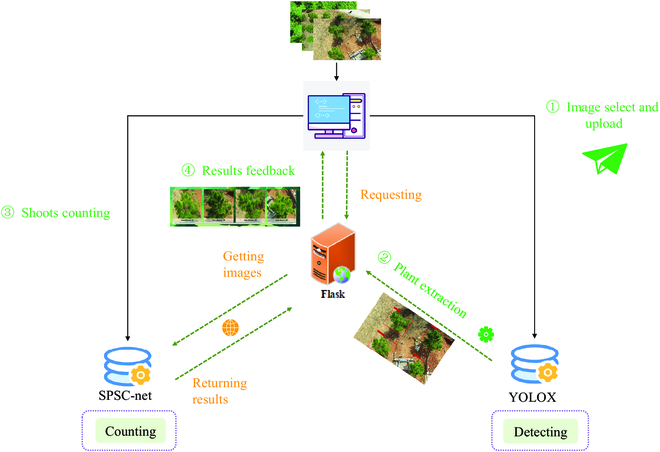
General flow chart of CountShoots.

The system was operated in “request-response” mode. The process of new shoots counting is depicted in Fig. 9. The specific process is as follows:

1. Image select and upload. The dialog function is used to obtain the image path. The image is read and displayed on the front-end webpage through the “imshow” function. The user clicks the "Upload" button to send the image to the server.

2. Plant extraction. This step is the first to be executed after the user triggers the “counting” task. The system updates the trained YOLOX model (Single-plant extraction network) to perform the canopy detection task and extracts the single plant according to the detection results. The detection results are feedback to the front-end webpage.

3. Shoots counting. This step is executed immediately after the second step. The system reads in the single-plant image obtained in the second step and then performs the trained counting model SPSC-net (New shoots counting model) to count the number of new shoots from the extracted canopy.

4. Results feedback. The final outcome (i.e., the counting results) is feedbacked to the user interface.

**Fig. 10.**
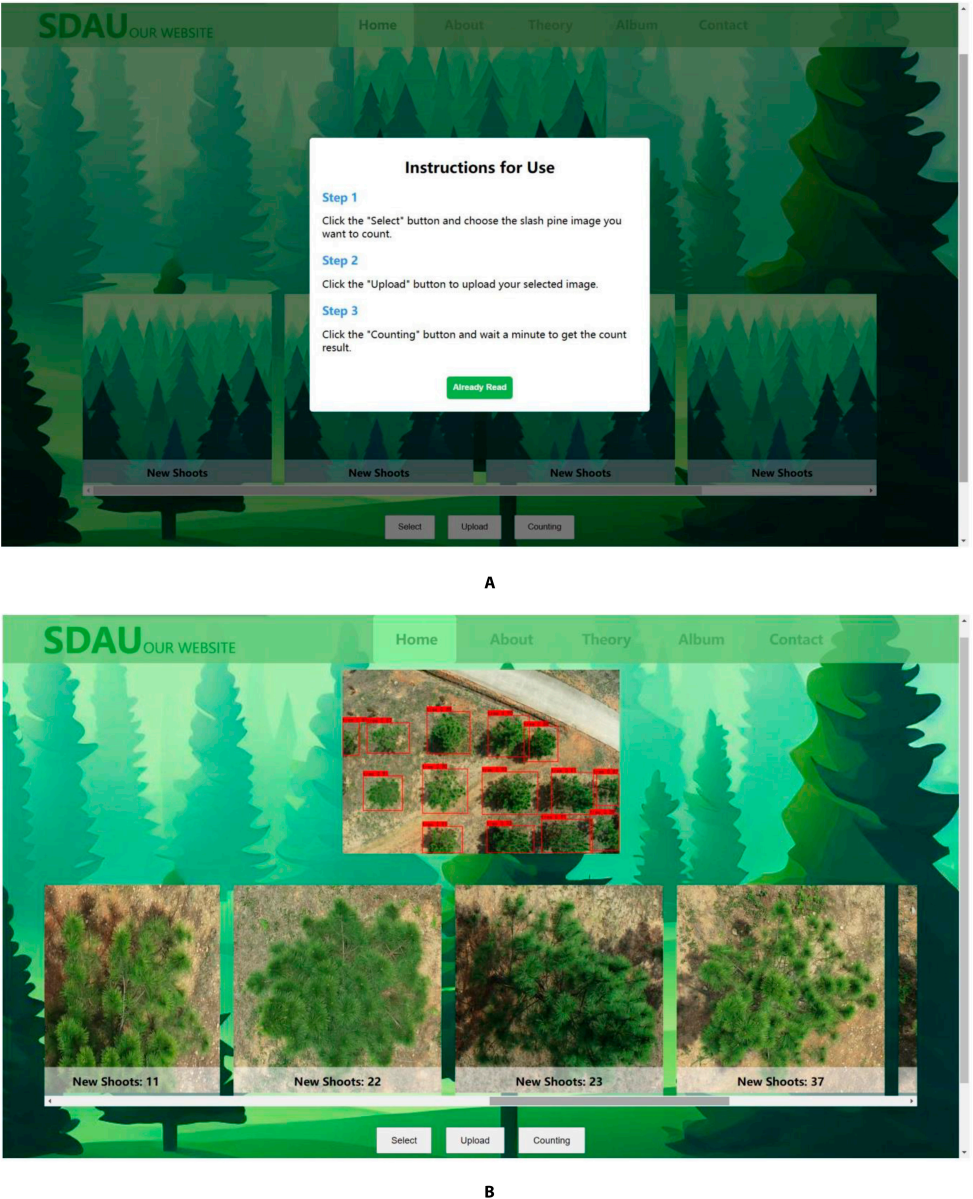
The counting interface of CountShoots. (A) Website usage instructions. (B) New shoots counting interface.

The counting interface of CountShoots is shown in Fig. 10. After entering the counting page, new users can follow the instructions on the homepage to perform counting tasks (Fig. 10A). In the new shoots counting interface, users can click the “Select” button to specify image path, click “Upload” button to load the slash pine image, and click the “Counting” button to count the number of new shoots. The users can directly view the number of new shoots in each crown, as shown in Fig. 10B.

## Discussion

In this study, the slash pine trees in a breeding population were employed for the development of tree identification and new shoots detection model using UAV imaging. The experimental results demonstrate the superiority of the proposed framework on multiscale image processing of slash pine. In addition, user-friendly software was created, which makes it more convenient and suitable for the tree breeding or ecological researchers to be used.

Firstly, we used a YOLOX model to identify the tree species and location from the complex background RGB images and compared it with other state-of-art published models. The results show that our model has the highest detection accuracy compared to YOLOv5, Efficientnet, and Faster-RCNN models. Similar results have been found in other object detection studies [48,49]. YOLOv5 showed less accuracy on the object detection than YOLOX model (Ge et al. [50], with values of 86.42% and 83.95% on object detection in the Beijing capital airport in China using the synthetic aperture radar imagery, but less than the spatial orientation attention enhancement network (90.12%). However, this result is lower than our study (i.e., recognition accuracy of 95.47% with a threshold equal to 0.75). Our YOLOX model produced a similar AP to the results of Wu et al. [51]. YOLOX and Tree Crown Detection network yielded accuracy rates of 96.30% and 96.76%, respectively, based on the identification of bayberry trees using UAV optical imagery. YOLOX model shows the best performance in the case of target coincidence, blurry and complex background. It innovatively employed the decoupled head and introduces the anchor-free detector to improve the performances of detection for the YOLOX model that can accurately find the overlap and small target of slash pine trees.

In the new shoot counting model, the newly developed counting network SPSC-net performed better than DM-Count, CSR-net, and MCNN models. SPSC-net uses the self-attention mechanism to capture the global features of the new shoots and designs the feature pyramid fusion module to supplement the details with the underlying features to obtain the extractive features rich in semantics, detail information, and global features. The hollow convolutional network was used in SPSC-net to better capture image information at different scales and predict the number of new shoots more accurately. In addition, in the SPSC-net, the unbalanced OT loss is designed to replace the OT loss in the CCTrans model. Firstly, the method directly takes the use of point-by-point loss and pixel loss to penalize the additional/missing density, while the original loss requires additional counting loss, which reduces counting efficiency. Secondly, the TV loss in the original loss is supervised pixel by pixel using the normalized dot plot, which is prone to overfitting. Through our experiment, we proved that the loss we used was more efficient at pushing density from the background to the annotation than the square Euclidean cost. The SPSC-net counting model performs better than the YOLOv5-SBiC model built by Liang et al. [43] which yields a recognition accuracy value of 79.6% on the detection of late-autumn shoots of Lichi trees, and the model built by Hong et al. [52] on the detection of wheat ear fusarium head blight based on RGB images, with an accuracy of 93.69%. However, it is important to note that this comparison serves as a rough overview and does not possess the same level of rigor as the main results.

It should also be mentioned that limitations occur in our model. Firstly, slash pine trees are a type of macrophanerophytes with tall crowns and luxuriant branches, and the new shoots at the lower layer may be shielded by the upper layers of the canopy, which cannot be captured by the UAV and detected by the models, making it difficult to accurately identify all the new shoots on the slash pine trees. The number of the lower layers of new shoots overlapped is much less, and most of these are small since they are at the early growth stage. Therefore, these have little effect on the total among new shoots. Secondly, the flying height of the UAV adopted to capture remote sensing images is strictly limited to 10 to 15 m. A higher flight height potentially reduces the detection accuracy due to the low resolution of remote sensing images. The impact of flight altitude and image resolution on the accuracy of new shoot counting was not investigated in this study, which could be another research topic for future studies. To acquire UAV imagery with limited flight height, it is time-consuming to survey for all trees in large orchards. Further testing is required for the practical applications of the model in other pine plantations.

To the best of our knowledge, our study developed a pipeline identifying tree crowns and counting new shoots using 2 separate models: the SPSC-net slash pine new shoots counting model and the tree crown detection network YOLOX. We obtained an accurate counting of new shoots of a single slash pine tree. The extraction network was implemented for the derivation and labeling of slash pine, reducing the influence of complex backgrounds such as soil and light. The counting network deployed used multivoid rate convolution to fuse multiscale features, increasing the accuracy of tip positioning by using nonequilibrium transmission and perspective-guided loss, generating a high-resolution density map of new shoots distribution, which provides a better supporting tool for forestry researchers. Meanwhile, an automatic new shoot counting system for slash pine called CountShoots was built, which efficiently and accurately estimated the number and distribution density of slash pine new shoots and provided reliable data support for subsequent genetic breeding and efficient breeding research of slash pine.

## Data Availability

The data in this study are available on upon request from the corresponding author. The counting system was publicly released at https://github.com/haohuihui5019/CountShoots.
